# An investigation of syrinx morphometry and sound frequency association in lovebirds ( *Agapornis fischeri*) chirps

**DOI:** 10.12688/f1000research.108884.3

**Published:** 2024-05-22

**Authors:** Cytra Meyliana Surya Dewi, Yeni Dhamayanti, Faisal Fikri, Agus Purnomo, Shafia Khairani, Shekhar Chhetri, Muhammad Thohawi Elziyad Purnama

**Affiliations:** 1 School of Health and Life Sciences, Universitas Airlangga, Surabaya, 60115, Indonesia; 2 Division of Veterinary Anatomy, Department of Veterinary Science, Faculty of Veterinary Medicine, Universitas Airlangga, Surabaya, 60115, Indonesia; 3 Division of Veterinary Clinical Pathology and Physiology, Department of Veterinary Science, Faculty of Veterinary Medicine, Universitas Airlangga, Surabaya, 60115, Indonesia; 4 Department of Veterinary Surgery and Radiology, Faculty of Veterinary Medicine, Universitas Gadjah Mada, Yogyakarta, 55281, Indonesia; 5 Department of Biomedical Science, Faculty of Medicine, Universitas Padjajaran, Bandung, 45363, Indonesia; 6 Department of Animal Science, College of Natural Resources, Royal University of Bhutan, Lobesa, Punakha, 13001, Bhutan

**Keywords:** Agapornis fischeri, biodiversity, lovebird, sound frequency, syrinx morphometry

## Abstract

**Background:**

In the issue of biodiversity, the domestication of birds as pets and trade animals requires special attention as a conservation effort. Lovebirds ( *Agapornis fischeri*) are popular birds worldwide, due to their varied ornamentation and melodic chirping sound. Syrinx structure is suspected to be the main source of sound production during the chirping period. This study aimed to investigate syrinx morphometry and its correlation with sound frequency produced in lovebirds.

**Methods:**

A total of 24 lovebirds of different ages and gender were investigated. Polymerase chain reaction method was performed to determine lovebird gender, meanwhile bird age was identified based on post-hatch recordings at the breeding farm. Thus, we enrolled male (n=12) and female (n=12) lovebirds aged 2 (n=4), 3 (n=4), and 4 (n=4) months in the investigation group, respectively. Fast Fourier Transform (FFT) was performed to evaluate sound frequency during chirping period. Then, syrinx morphometry was identified using a topographic approach and methylene blue staining. Each variable was evaluated with Image J software and vernier caliper.

**Results:**

Based on a topographical approach, we reported the general cartilage structure of the tracheosyringeal, bronchosyringeal, paired protrusions, tracheolateral muscles, sternotracheal muscles, and syringeal muscles in lovebird syrinx. In particular, the tympaniform membranes lateral lead a crucial role in modulating the frequency of male lovebirds more significantly (p=0,009) compared to female. On the other hand, the tympaniform membranes lateral dexter (p=0,02) and sinister (p=0,05) in females showed wider compared to male. We also reported a negative correlation between sound frequency compared to tympaniform membranes lateral dexter (y = -913,56x + 6770,8) and sinister (y = -706,16x + 5736).

**Conclusions:**

It can be concluded that the tympaniform membranes lateral produced the lovebirds’ primary sound. The sound frequency of male lovebirds was higher compared to female, however negatively correlated with the area of tympaniform membranes lateral.

## Introduction

In the last decade, various birds have been domesticated as pets. In Indonesia, lovebirds ( *Agapornis fischeri*) are popularly reared in captivity and are traded pets. Lovebird is an attractive ornamental bird because it has a characteristic feather color pattern and distinctive behavior. In addition, the sound of birds chirping and variations in sound can attract the attention of breeders to rear lovebirds. ^1^ Lovebird is a small parrot that often participates in chirping contests, thereby increasing its economic value as a traded pet. In Indonesia, the lovebird contests are organized by enthusiastic bird communities to evaluate the criteria for plumage color and chirp production. However, the risk of bird population sustainability must be a concern ^2^

In some groups of avians, the syrinx is a crucial organ for producing sound and melody during chirping. ^3^ The chirping variation is a manifestation of the articulation formed by the syrinx cartilage. In previous studies, syrinx organs were morphometrically revealed in pigeons, ^4^ parakeets and canaries, ^5^ chickens and rock dove, ^6^ sparrows ^7^ and penguins. ^8^ The syrinx will stimulate the vibrations reflected from each cartilage to produce sound frequency. ^9^ Lovebirds voice articulation develops in five phases from 0 to 91 days old, thereafter lovebirds can produce clear sound with different configurations. The duration of the sound and chirping frequency produced by the syrinx is significantly increased due to the reflection of the tympaniform membrane lateral. ^10^

A pair of tympaniform membrane lateral are found in the lateral wall of the syrinx at the tracheobronchial junction. This structure extends from the tympanum to the first bronchial ring in chickens and pigeons or is composed of short membranes between the bronchosyringeal rings. ^11^ The tympaniform membrane medial connects the pessulus to the medial portion of the primary bronchus. This structure connects the ring portion of the bronchosyringeal cartilage dorsoventrally. ^12^ The labia are a pair of elastic cords found in the lateral and medial tympaniform membranes that physiologically produce the primary sound. ^13^

The difference between male and female lovebirds requires further information, in particular, to determine the eligible age for the Lovebirds contest. Age may influence syrinx morphometry and normal function of the tympaniform membrane lateral to produce sound. In addition, it is very important to investigate the association of syrinx morphometry and sound frequency. This study was expected to provide an evidence of syrinx topography and morphometry in lovebirds and their association on the sound frequency produced with differences in gender and age.

## Methods

### Ethics statement

This study was approved by the Ethical committee of animal care and use, Universitas Airlangga (No.445/HRECC.FODM/X/2020). This study was also reported according to the Animal Research: Reporting of in vivo Experiments (ARRIVE) guidelines 2.0: author checklist (see *Reporting guidelines* ^52^). All efforts were made to ameliorate any suffering of animals.

Based on legislation from the Ministry of Forestry and Biodiversity, Republic of Indonesia with reference number (No.20/2018) and Government Regulation with reference number (No.8/1999), Fischer’s Lovebird is not included in the red list in Indonesia. In addition, the owners of the rearing farm held special conservation permission and is reported periodically with an increase in the Fischer’s Lovebird population at the rearing farm.

### Study period and location

This study was conducted over five months (November 2020 - March 2021). These lovebirds were reared in Mr. Sutoro farm, Solo, Central Java, Indonesia (7°33′29.6″S 110°51′43.0″E). Sound frequencies evaluation and syrinx morphometry were investigated at the biology laboratory, Universitas Surakarta.

### Experimental design

The sample size was determined using Federer’s formula: t(n-1) ≥ 15 with a combination of age and gender for six groups. This formula design was considered due to two aspects of the same thing i.e. randomization and replication are necessary to obtain a valid estimate of the error variance of a contrast. ^14^ There were four replications in each group, meaning a total of 24 lovebirds of different ages and gender (12 males and 12 females) were investigated. Inclusion criteria were that the birds were healthy (no animals were excluded). These lovebirds were reared in a fenced enclosure, fed millet seeds, and fresh water ad libitum. Our study was conducted on 2, 3, and 4-month-old (mo) birds based on post-hatch recordings at the breeding farm.

**Sex determination in lovebirds**

Meanwhile, sexing was performed on feather samples using the polymerase chain reaction (PCR) method. Deoxyribonucleic acid (DNA) isolation was performed using the Gsync DNA Extraction Kit protocol (Geneaid Biotech Ltd, Taiwan). DNA extraction was carried out overnight to produce clear DNA bands. DNA fragment amplification was carried out targeting the CHD gene using primers and reference primary markers ( Table 1). The reaction mixture of bird DNA was mixed as much as 25 μl consisting of 12,5 μl MyTaq™ DNA Polymerase (Labgene Scientific SA, Switzerland), 1 μl forward primer, 1 μl reverse primer, and 9,5 μl isolated DNA. Thereafter, the mixture was incubated in a PCR machine followed by pre-denaturation at 94°C for 2 mins, denaturation at 94°C for 20 sec, annealing at 46°C for 30 sec, extension at 72°C for 40 sec, and final extension at 72°C for 10 mins. All stages were repeated for 40 cycles. The results of the electrophoresis were then determined by comparing all samples with a 100 bp HyperLadder™ marker (Bioline, UK). The results of the electrophoresis are available in *Underlying data*. ^49^ Visualization was revealed PCR products of 400 bp and 350 bp for females, meanwhile only 400 bp for males. Of the 33 collected samples, only male (n=12) and female (n=12) lovebirds aged 2 (n=4), 3 (n=4), and 4 (n=4) mo were enrolled in the investigation group, respectively. Randomization and blinding of the collected samples were performed in replicated gender and age groups using the following steps: (1) Lovebird populations aged 2, 3, and 4 months in the respective fenced enclosures were assigned as the investigated group, (2) lovebirds were caught one by one randomly and then their gender was determined using the PCR method, (3) only 4 male and 4 female lovebirds in the respective age were enrolled and labeled as samples, (4) this method was repeated for the 3 and 4 month age groups.

**Table 1.** The primary sequence of nucleotides in this study.

Code	Nucleotide	Primer
NP-F ^15^	5’-GAGAAACTGTGCAAAACAG-3’	Forward
P2-R ^15^	5’-TCTGCATCGCTAAATCCTTT-3’	Reverse
MP-R ^16^	5’-AGTCACTATCAGATCCGGAA-3’	Reverse

### Fast Fourier Transform (FFT) method

We evaluated each bird separately. The Fast Fourier Transform (FFT) ^17^ method was performed to record the lovebirds’ sound frequency in an insulation room to avoid noise. The analog recording process was performed in an interval of 30 mins to record the number of bird sounds. Analog data on the recording device was transferred to a computer using a soundcard and then converted into WAV (Microsoft Waveform Audio Files) format files using Microsoft Sound Recording software ( Windows Voice Recorder for Windows 10, Microsoft Corporation) (see *Underlying data* ^50^). The visualization of voice phrases described the characteristics of the sound in a time interval in the form of a spectrogram (see *Underlying data* ^51^). In addition, the visualization of sound phrases was observed as a continuous wave to reduce errors due to noise and phase differences in the FFT method.

### Methylene blue staining ^18^

Euthanasia was performed by injection of a lethal dose (LD50) of 99.5 mg/kg BW of ketamine intramuscularly then observed for 8-15 mins. ^19^ The dissection procedure was performed on the dorsal recumbency with a focus on the thoracic cavity and then the topography of the dorsal, ventral, and lateral site of the syrinx was observed. The lower respiratory organs were dissected and then stored for 2 × 24 h using 10% formalin solution, then as a stopping point, moved the organs into 70% alcohol solution. Syrinx organs were immersed in 1% methylene blue for 15 mins and then rinsed using 50% and 70% alcohol.

### Syrinx morphometric evaluation

Topographical anatomical observations of the syrinx were performed for the following variables: tympanum (Ty), processus tympanicus (PT), tracheosyringeal (TS), bronchosyringeal (BS), bronchus (B), trachea bifurcation (BT), tracheolateral muscle (TLm), sternotracheal muscle (STm), profundal syringeal muscle (SPm), and superficial syringeal muscle (SSm). Meanwhile, syrinx morphometric were evaluated for the following variables: tympaniform membrane lateral dexter (TMLd), tympaniform membrane lateral sinister (TMLs), upper trachea (upT), middle trachea (mT), lower trachea (loT), paired protrusions dorsal (PPd), and paired protrusions ventral (PPv). Each variable was observed using a stereo microscope and then evaluated using Image J version 1.8.0 software. ^20^

**Figure 1.**
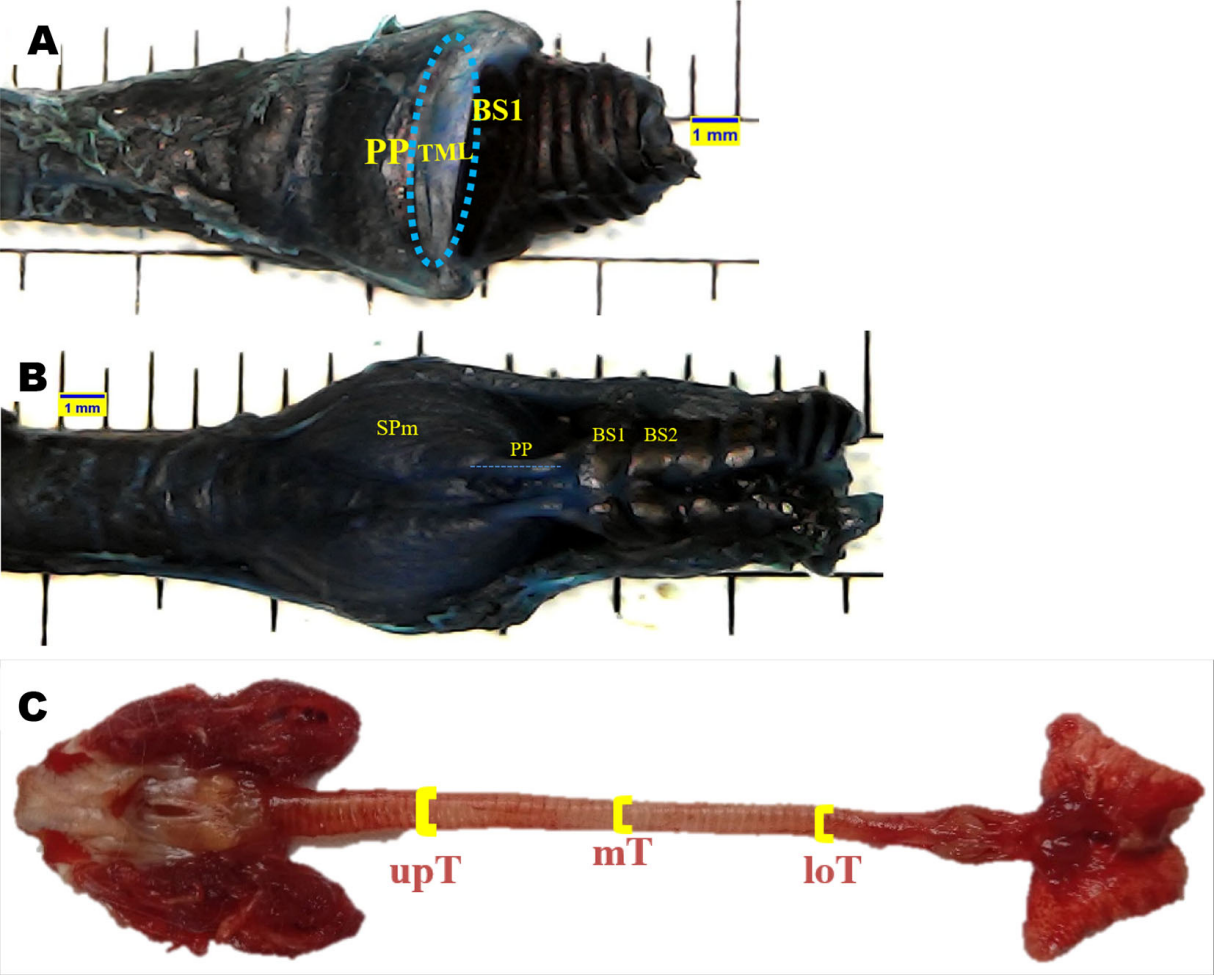
Syrinx morphometric determination. (A) area of tympaniform membrane lateral (TML), (B) length of paired protrusions (PP), (C) trachea diameter.

The area of tympaniform membrane lateral dexter and sinister were determined according to the curved line around the lateral side of the tympaniform membrane lateral. The cranial and caudal margins of the tympaniform membrane lateral are the paired protrusions and bronchosyringeal cartilages ( Figure 1A). ^4^ The length of the paired protrusions dorsal and paired protrusions ventral were determined according to the cranial to caudal line. This line is emphasized from the end profundal syringeal muscle to the bronchosyringeal-1 cartilage ( Figure 1B). ^5^ For trachea diameter, evaluation was performed on the upper trachea, middle trachea, and lower trachea sides using a vernier caliper ( Figure 1C).

### Statistical analysis

The normality of the data was analyzed using the Shapiro–Wilk test with a normality value of p > 0,05. All the data were expressed as mean ± standard error (SE) then analyzed using multivariate analysis of variance (MANOVA) followed by Duncan’s comparison test. Values were considered significantly different at p < 0,05. The sound frequency of male and female birds were analyzed using independent T-test with a significance level (p < 0,05). Meanwhile, to investigate the association between the area of tympaniform membrane lateral and the lovebirds sound frequency produced, an association test was performed. Statistical analysis was performed using SPSS v.25 software (IBM, USA).

## Results

### Topographic anatomy and morphometry of the syrinx

**Figure 2.**
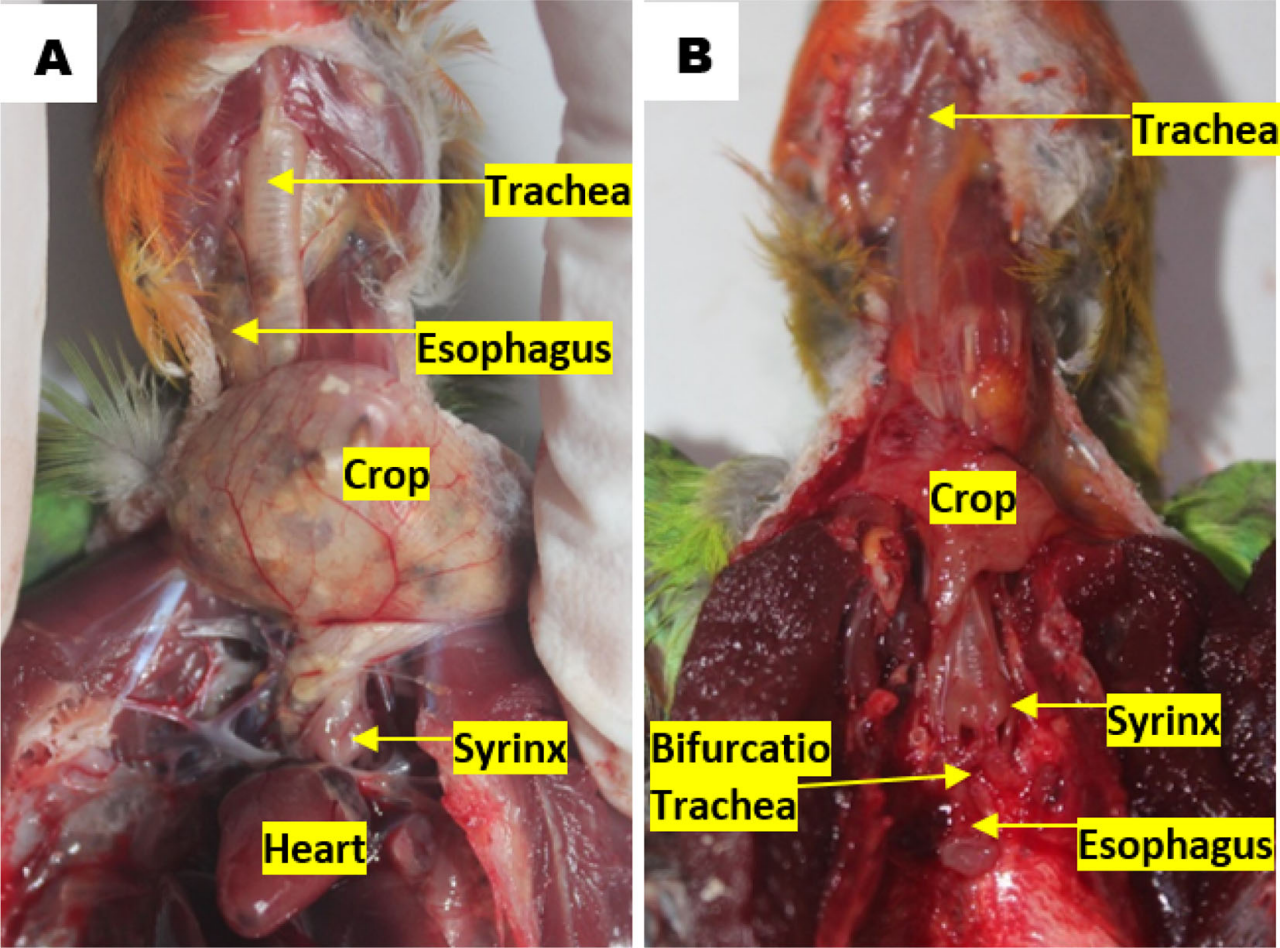
Location of the syrinx to (A) the heart and (B) the esophagus in the thoracic cavity.

The raw, uncropped images associated with syrinx morphometry and topography are available in *Underlying data*. ^48^ The topographical observations of lovebird syrinx showed that there was no anatomical topographic variation across the various age and gender groups. Syrinx location in the thoracic cavity tends to the left side compared to the esophagus, on the dorsocranial of the heart and caudal of the crop. Tracheal organs were found in the ventral esophagus. The trachea in the cervical region was found on the left side of the esophagus, before the thoracic cavity of the trachea is on the dorsal side of the esophagus. The crop continues to the caudal side of the transformed esophagus parallel to the syrinx. The esophagus continues to the caudal side by crossing to the dorsal bifurcation of the trachea then being on the ventral of the lung and transformed into the proventriculus ( Figure 2).

**Figure 3.**
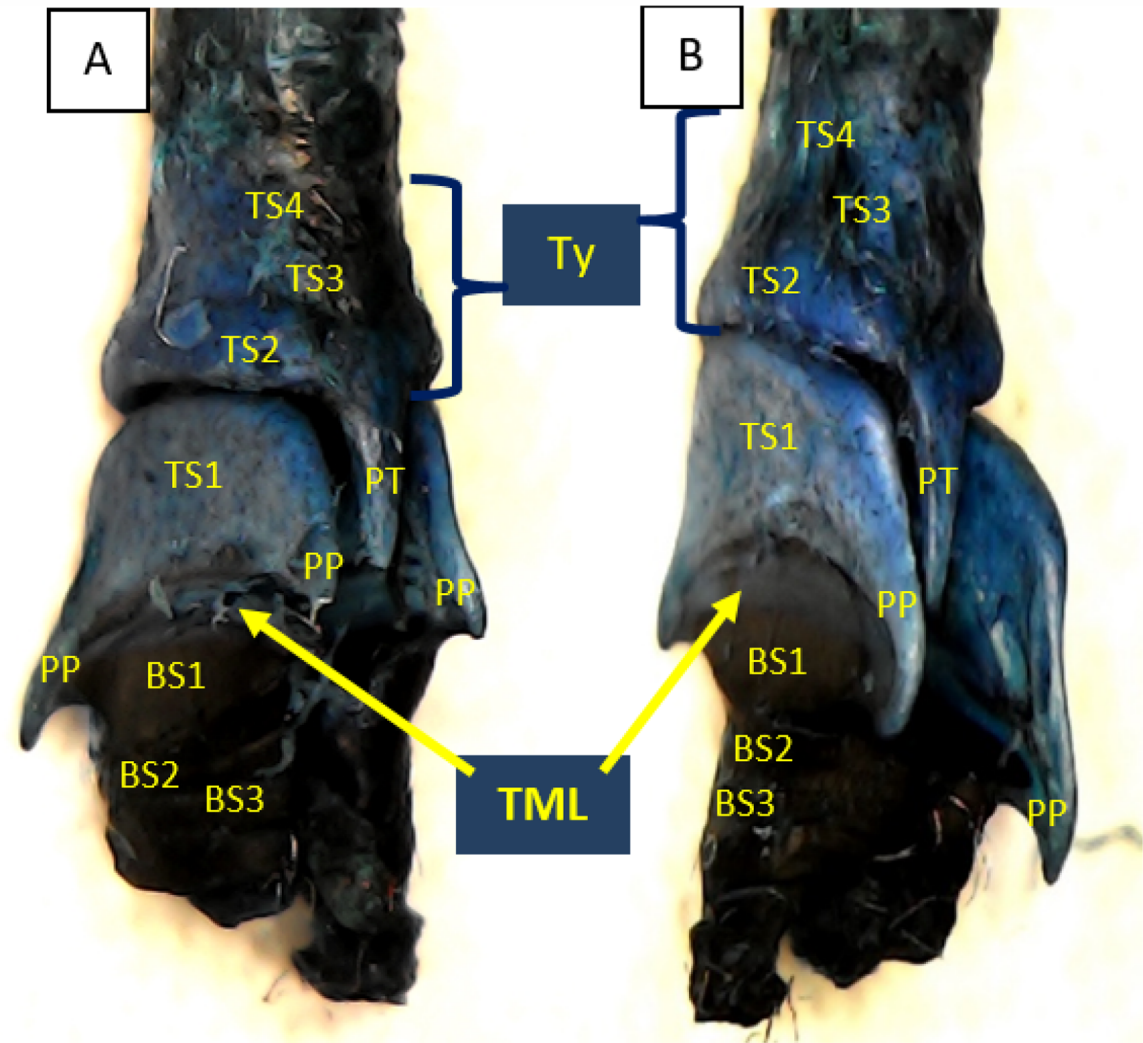
Syrinx structures with methylene blue staining (A) dorsolateral view and (B) ventrolateral view. TML= tympaniform membrane lateral, Ty = tympanum, PT = processus tympanicus, PP = paired protrusions, TS1-4 = tracheosyringeal 1-4, BS1-3 = bronchosyringeal 1-3.

**Figure 4.**
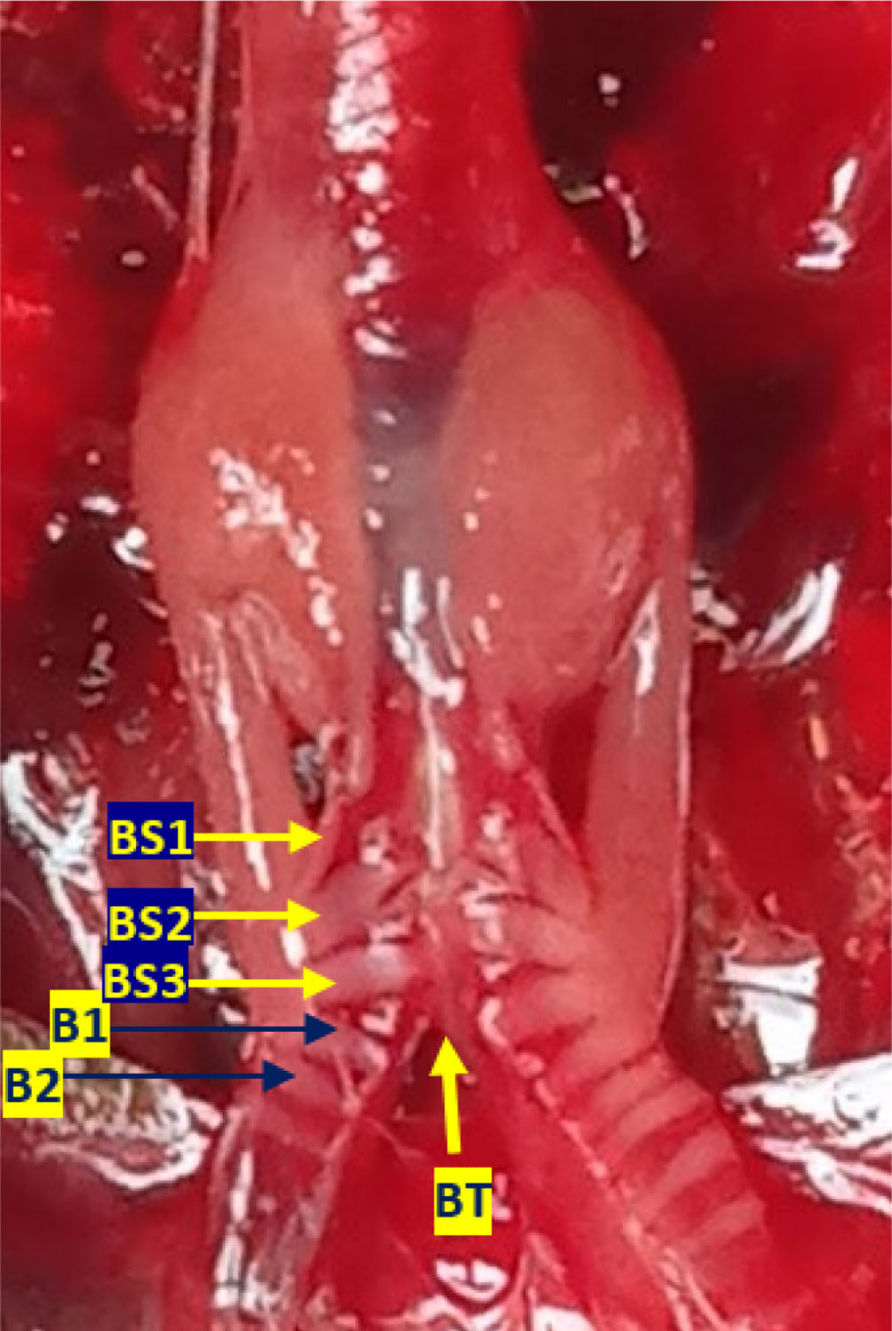
The structure of the syrinx cartilage. BS1-3 = bronchosyringeal 1-3, B1-2 = bronchus 1-2, BT = trachea bifurcation.

Using methylene blue staining, clearly visible tympaniform membrane lateral was observed from both primary bronchi. The cranial margin of the tympaniform membrane lateral is paired protrusions covered by profundal syringeal muscle and the caudal margin of the tympaniform membrane lateral is the bronchosyringeal-1 cartilage. An evidence revealed that pessulus and labia were not found in the lovebird syrinx. It was found tympanum modification in the form of caudal protrusion called processus tympanicus on the dorsal and ventral sides. Processus tympanicus structure was found between a pair of paired protrusions which are tympanic plates and are connected to the cranial side of the tympaniform membrane lateral. Paired protrusions and tympanum structures are part of the tracheosyringeal cartilage. Paired protrusions is tracheosyringeal-1 cartilage, meanwhile tympanum is composed of three cartilages i.e. tracheosyringeal 2-4 ( Figure 3). Bronchosyringeal cartilage was found on the cranial side of the trachea bifurcation and the caudal side of the tympaniform membrane lateral. All samples from each gender and age group had three pairs of bronchosyringeal cartilage on the right and left sides ( Figure 4).

**Figure 5.**
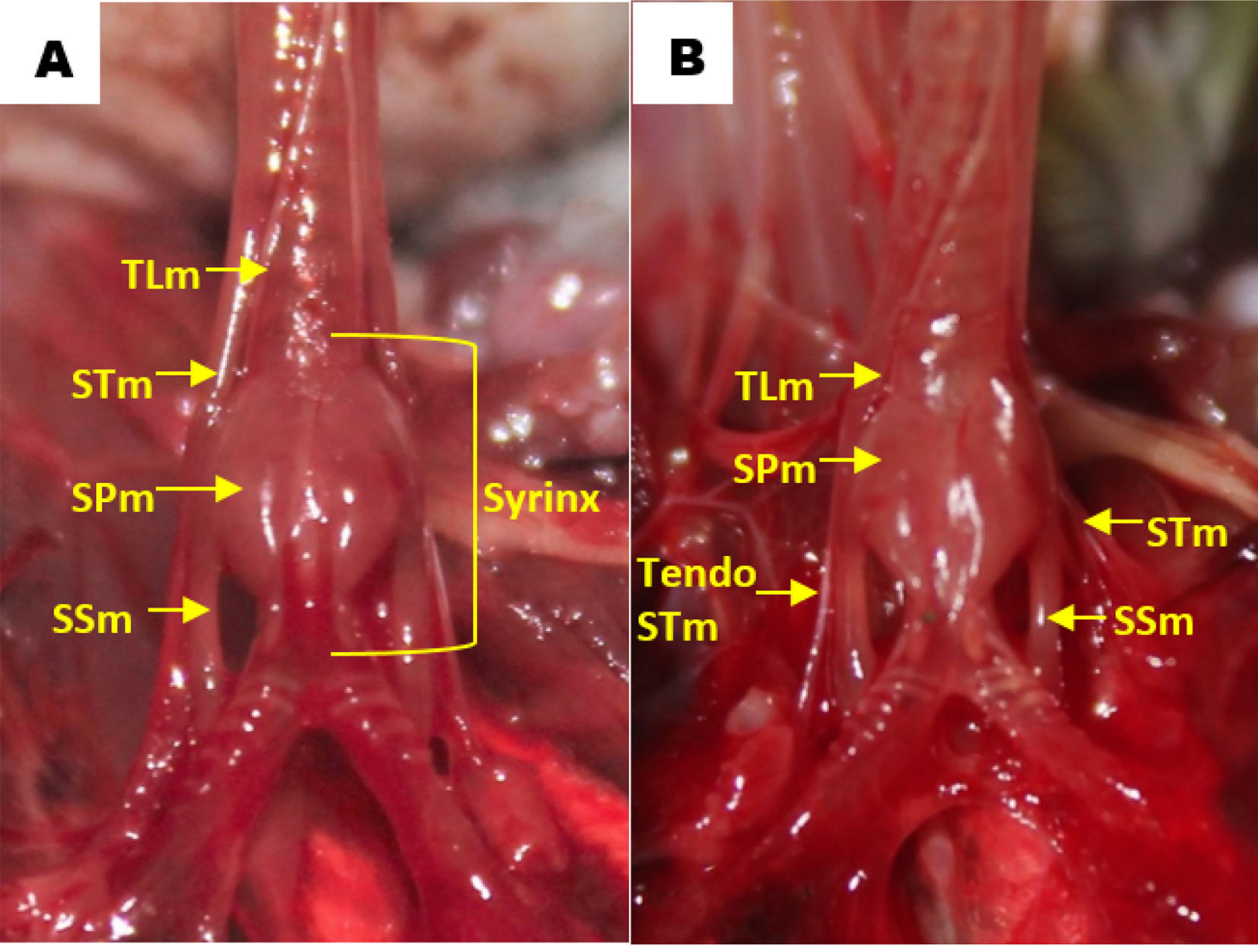
Muscle structures in the syrinx (A) dorsal view and (B) ventral view. TLm = tracheolateral muscle, STm = sternotracheal muscle, SPm = profundal syringeal muscle, SSm = superficial syringeal muscle.

**Figure 6.**
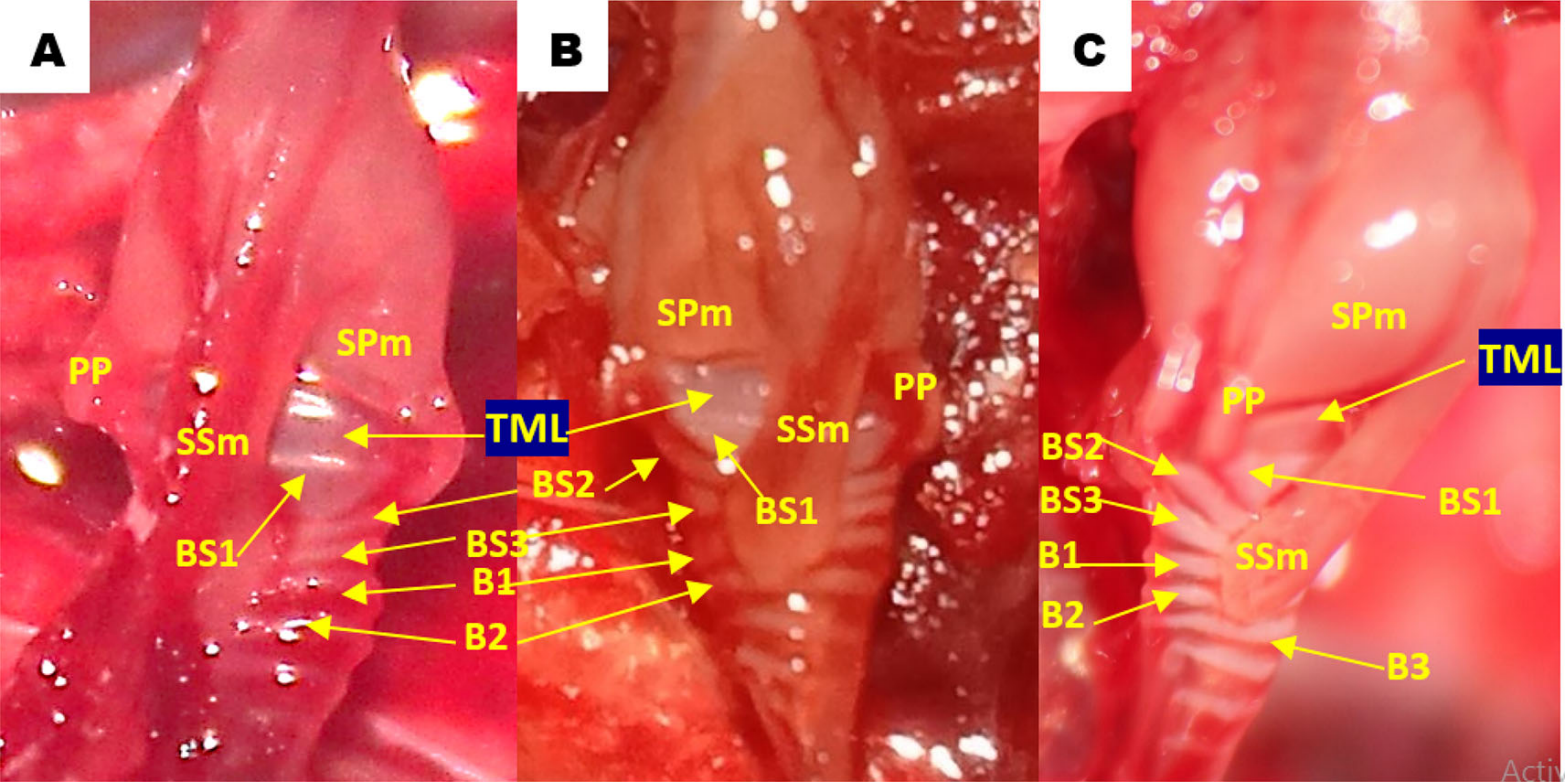
Lateral view of the lovebirds syrinx aged (A) 2 months old (mo), (B) 3 mo and (C) 4 mo. TML = tympaniform membrane lateral, PP = paired protrusions, SPm = profundal syringeal muscle, SSm = superficial syringeal muscle, BS1-3 = bronchosyringeal 1-3, B1-3 = bronchus 1-3.

Observation of the muscles revealed that in all gender and age groups the lovebird syrinx was identified as tracheolateral muscle, sternotracheal muscle, profundal syringeal muscle and superficial syringeal muscle. Tracheolateral muscle was found on the lateral side of the trachea. Meanwhile, sternotracheal muscle was found on the lateral side of the lower trachea and then split into two in the dexter and sinister syrinx ( Figure 5). The muscular structure gets thinner on the mediastinal side and eventually forms the sternotracheal muscle tendon, which then attaches to the lungs and extrapulmonary bronchi. Sternotracheal muscle on both sides looks asymmetrical, however the sinister is more inclined to the dorsal compared to the dexter is inclined ventrally. Profundal syringeal muscle was found from tympanum to paired protrusions with the caudal end of the muscle connected to the cranial portion of the tympaniform membrane lateral. Superficial syringeal muscle was identified from the lateral side of tympanum to the primary bronchus. The difference was found in the location of the connection of the caudal end of the profundal syringeal muscle and the bronchial cartilage. In groups aged 2 and 3 mo, both male and female, it was reported that there was a connection of the caudal end of the profundal syringeal muscle with the bronchus-2 cartilage, meanwhile, in the aged 4 mo group, both male and female, it was found in the bronchus-3 cartilage ( Figure 6).

In this syrinx morphometric study, it was reported that the area of the tympaniform membrane lateral dexter and sinister increased with the increasing age of lovebirds. Tympaniform membrane lateral dexter and sinister in female lovebirds were observed more widely compared to male lovebirds. In the paired protrusions variables, upper, middle, and lower tracheal diameters, there was no significant difference reported in gender and age groups ( Table 2). The full data associated with the Syrinx morphometry and sound frequency evaluation is available in *Underlying data*. ^47^

**Table 2.** Syrinx morphometric investigation of lovebirds aged 2, 3, and 4 months old (mo).

Variables	Male (n = 12)			Female (n = 12)			p-value between gender
	2 mo (n = 4)	3 mo (n = 4)	4 mo (n = 4)	2 mo (n = 4)	3 mo (n = 4)	4 mo (n = 4)	
Area of TMLd (mm ^2^)	1,81 ± 0,14 ^a^	2,89 ± 0,09 ^b^	3.75 ± 0,20 ^c^	1,38 ± 0,55 ^a^	3,74 ± 0,21 ^b^	4,33 ± 0,13 ^c^	0,02*
Area of TMLs (mm ^2^)	1,58 ± 0,11 ^a^	2,64 ± 0,23 ^b^	3,56 ± 0,23 ^c^	1,19 ± 0,19 ^a^	3,27 ± 0,15 ^b^	3,90 ± 0,19 ^c^	0,05*
Diameter of upT (mm)	2,53 ± 0,29	2,83 ± 0,20	2,50 ± 0,30	2,88 ± 0,08	2,47 ± 0,35	2,91 ± 0,20	0,29
Diameter of mT (mm)	2,40 ± 0,10	2,33 ± 0,18	2,24 ± 0,23	2,47 ± 0,32	2,37 ± 0,23	2,58 ± 0,13	0,17
Diameter of loT (mm)	2,75 ± 0,23	2,35 ± 0,31	2,00 ± 0,09	2,52 ± 0,38	2,60 ± 0,35	2,48 ± 0,38	0,27
Length of PPd (mm)	2,39 ± 0,06	2,48 ± 0,15	2,47 ± 0,26	2,64 ± 0,09	2,50 ± 0,14	2,58 ± 0,35	0,21
Length of PPv (mm)	2,88 ± 0,19	3,06 ± 0,33	3,34 ± 0,24	3,25 ± 0,35	2,95 ± 0,11	3,42 ± 0,38	0,41

Values are expressed in mean ± SE (n = 4 samples for each different gender and age groups). Multivariate analysis of variance was carried out followed by Duncan’s comparison test. ^a,b,c^ different superscripts in the same row indicate significant differences (p < 0,05). TMLd = tympaniform membrane lateral dexter, TMLs = tympaniform membrane lateral sinister, upT = upper trachea, mT = middle trachea, loT = lower trachea, PPd= paired protrusions dorsal, PPv = paired protrusions ventral.

### Sound frequency evaluation

**Figure 7.**
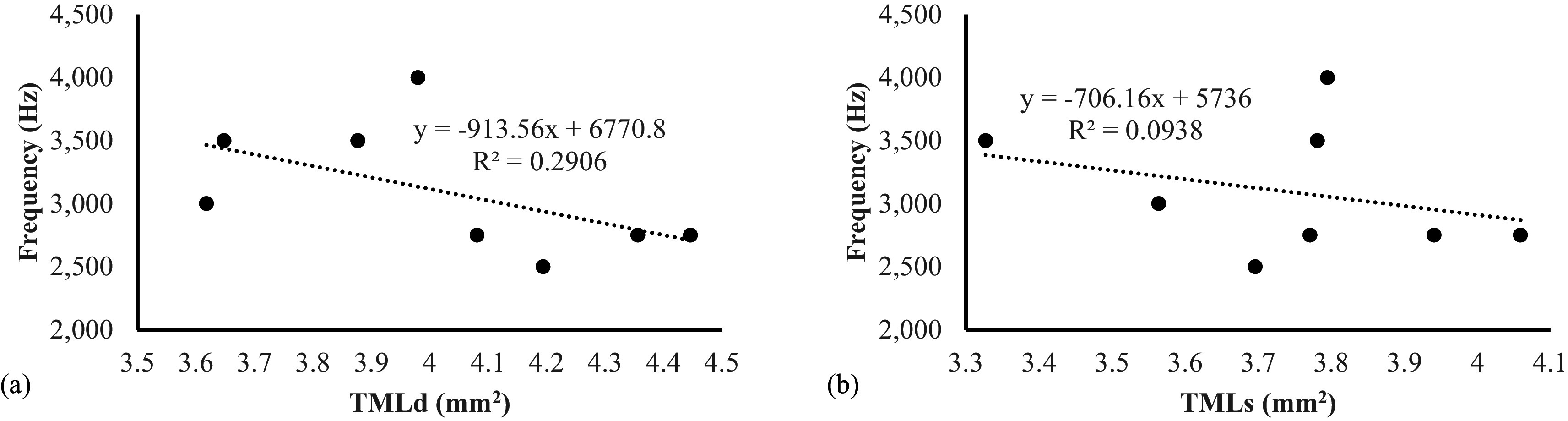
Association between lovebirds sound frequencies on (a) TMLd = tympaniform membrane lateral dexter (b) TMLs = tympaniform membrane lateral sinister.

In this present study, the evaluation of sound frequency was performed only on lovebirds aged 4 mo because at that age they are considered a mature lovebird during contest. The sound frequency produced by male lovebirds (3500,00 ± 204,12) was significantly higher compared to female lovebirds (2687,50 ± 62,50) ( Table 3). This study also revealed that there was a negative association between sound frequencies compared with the area of tympaniform membranes lateral dexter (y = -913,56x + 6770,8) and sinister (y = -706,16x + 5736) ( Figure 7). This finding indicated that the smaller area of lateral tympaniform membranes followed by an increase in the sounds frequency produced by the syrinx cartilage articulation.

**Table 3.** Sound frequency of male and female lovebirds aged 4 months old.

Gender	Peak frequency	Interval (sec)	Frequency (Hz)	Mean ± SE	p-value for frequency
Male (n = 4)	2	0,1	3500	3500,00 ± 204,12	0,009**
	2	0,1	3000		
	1	0,3	4000		
	1	0,2	3500		
Female (n = 4)	2	0,1	2750	2687,50 ± 62,50	
	2	0,2	2750		
	1	0,1	2500		
	2	0,2	2750		

Independent T-test was carried out with a significance level (p<0,05).

## Discussion

Modification of the syrinx in male birds is related to dimorphism of muscle mass and nerve fiber components in the laryngeal canal. Sexually dimorphic vocal behavior arises from each vibration reflected by the pesulus and often represents a more significant evolution in bird vocal complexity. This adaptation illustration allows the potential to generate an enhanced frequency range. ^21^ The syrinx is a vocal organ in the avian family with the physiological principle of flowing air from the lungs across the narrow bronchotracheal tract and vibrating the tympaniform membranes lateral and medial. ^22^ In respective bird species there is a variety of sounds due to differences in the syrinx anatomical structure. ^23^ In this present study, we observed a modified syrinx cartilage in the form of processus tympanicus and paired protrusions which are only owned by the Psittacidae bird family. ^24^ Paired protrusions are classified into tracheosyringeal cartilages with a composition similar to the tympanum compared to the structure of the bronchosyringeal cartilages. ^25^

In a previous study involving male Budgerigars ( *Melopsittacus undulatus*), the tympanum was composed of six elements, ^26^ meanwhile in this study the tympanum was only composed of three elements. Lovebird syrinx was found on the caudal side of the lower trachea and not yet connected to the trachea bifurcation. The syrinx type in the Psittacidae family has a single set of membranes so that it produces only a single sound, unlike other bird species with the tracheobronchial syrinx type and can produce multiple sounds independently. ^27^ The sternotracheal muscle was identified from the lower trachea and then transformed into a tendon that connects to the lung area near the end of the extrapulmonary bronchus called the septum obliquum. ^28^

In another study, the sternotracheal muscle appeared asymmetrical because the syrinx crossed with the esophagus, thus the muscle junction was not parallel between the dexter and sinister sides. ^29^ Another characteristic of the Psittacidae family, it has the Syringeal profundus and superficial muscle which is found in all gender and age groups with insertions starting from the cranial tympanum. Lovebirds only have two intrinsic muscles, i.e. the syringeal profundus and superficial muscles compared to other species of chirping birds due to the muscle structure is more complex, i.e. the tracheobronchial dorsal, ventral and brevis muscles and the syringeal dorsal and ventral muscles. ^30^

In this syrinx morphometric study, there were significant differences in the area of tympaniform membranes lateral dexter and sinister in lovebirds aged 2, 3, and 4 mo. The area of the tympaniform membranes lateral increases with age in both male and female lovebirds. Tympaniform membranes lateral are functional membranes that produce melodic sounds in the Psittacidae family. ^31^ The development of the tympaniform membranes lateral is triggered by the development of the chirping sound. The rapid development that occurs in pubertal birds will then decrease gradually at aged 9 mo. ^32^

In another study, tympaniform membranes lateral were reported to be larger in male pigeons than in female pigeons. ^33^ This is supported by studies of the duration and number of chirping periods being greater in females than males. Studies on artificial vocal cords reported that the larger area of the vibrating membrane produces lower sound frequencies. ^34^ The area of the tympaniform membranes can be one of the factors that trigger the difference in the sound of chirping in male and female lovebirds. In addition, the body weight of female lovebirds is significantly heavier than males. Generally, bird species with larger bodies have lower sound frequencies. ^35^ Hormonal factors also affect the character of the sound produced. ^36^ Repeated administration of testosterone in perdix birds resulted in a longer duration of chirping with a lower frequency. ^37^ The same study stated that birds treated with testosterone showed a significant widening of the lumen of the trachea and bronchi and a thicker syrinx membrane. ^38^

Tympaniform membranes lateral in chickens are composed of connective tissue and an extracellular matrix consisting of carbohydrates, hyaluronic acid and collagen. ^39^ The vocal folds are sensitive to the hormone estrogen. The decrease in estrogen has an impact on changes in the expression of molecules in the extracellular matrix, whereas testosterone does not affect the extracellular matrix. ^40^ Studies on sparrows after hatching at 3, 10 and 17 days old showed that androgen receptors can be found in the muscles around the syrinx and estrogen receptors are found in many syrinx chondrocytes. ^41^ Estrogens and androgens can affect the development of the syrinx of birds after hatching in the skeletal and muscular parts, but their effect on the differentiation process is unidentified. Hormones have an influence on the nervous system which controls sound production. Testosterone has two effects, i.e. anatomically controlling the growth of vocal control stations and physiologically controlling the number of enzymes required during neural transmission. ^42^

In another study evaluated Anna’s hummingbird, the feather’s flapping at its resonance frequency creates the sound. The investigations showed that the R5’s trailing vane is the source of the sound, indicating that the resonance is related to the feather’s mechanical characteristics. Both flags and wind instruments’ reeds are examples of structures whose elastic modulus and shape cause them to vibrate in fluid flow at a resonant frequency. The R5 feathers feature a critical fluttering velocity as well as a critical minimum velocity for sound creation. The frequency that R5 produces varies very little with wind velocity above the critical velocity. ^43^ Meanwhile, the Mourning Dove ( *Zenaida macroura*) produces a tone that seems to be generated by the wings in a mechanical way. This sound is mostly heard during takeoff, which could be because of the high wingtip velocity brought on by the kinematics of takeoff. The primary means of modulating local air velocity to surpass a feather’s critical velocity for other feathers, including tail feathers, is to alter flight speed. Birds can make a wide variety of sounds, and non-vocal techniques like this tail feather sonation increase that diversity. The size of their syrinxes may restrict the volume of vocalizations that little birds may make. This implies that it has been able to overcome the inherent limitations on vocal sound volume by converting to feather sonations. ^44^

Vocal control stations on the central nervous system have androgen and estrogen receptors that affect sound sensitivity. ^45^ Studies on adult canaries require estrogen to produce a chirp with a high rate of repeating syllables. ^46^ The duration of the chirp, the length of the chirping element and the frequency range can be influenced by the hormone testosterone but only in certain species so that there are differences in the expression of hormone receptors in the central nervous system which differ in each species and even gender. Further investigation is required to determine how the anatomical and morphometric variations of the syrinx, light-dark cycles, breeding cycles, and seasonal changes, relate to the features of the chirping noises generated. Furthermore, it is strongly advised to compare the sound intensities recorded during the study with the publicly available Xeno-Canto World Wild Bird Sounds collection in order to manage bird classification records and deliver quick, real-time information for the aim of determining biodiversity. Furthermore, the problem of urbanization today presents significant obstacles for animal communication, meaning that the singing habits of birds depend on both urban ecology and human activities.

## Conclusions

In conclusion, we identified tympanum, processus tympanicus, tracheosyringeal cartilage, bronchosyringeal, bronchi, trachea bifurcation, paired protrusions, tracheolateral muscle, sternotracheal muscle, profundal and superficial syringeal muscle in lovebird syrinx. Our study emphasized that tympaniform membranes lateral initiates the lovebirds’ primary sound, specifically in male birds. This finding also highlighted that the narrower tympaniform membranes lateral reflects the enhanced frequency of chirping in a single vocal period.

## Data availability

### Underlying data

Figshare: Syrinx morphometry and sound frequency evaluation. https://doi.org/10.6084/m9.figshare.18382925.v4. ^47^

This project contains the following underlying data:

• Syrinx morphometry and sound frequency evaluation.xlsx

Figshare: Syrinx morphometry and topography. https://doi.org/10.6084/m9.figshare.18386744.v2. ^48^

This project contains the following underlying data:

• 1a.png (Figure 1A Tympaniform membrane lateral evaluation).
• 1b.png (Figure 1B Paired protrusions evaluation).
• 1c.png (Figure 1C Trachea evaluation).
• 2a.png (Figure 2A Location of the syrinx compared to the heart in the thoracic cavity).
• 2b.png (Figure 2B Location of the syrinx compared to the esophagus in the thoracic cavity).
• 3a.png (Figure 3A Syrinx structures with methylene blue staining on dorsolateral view).
• 3b.png (Figure 3B Syrinx structures with methylene blue staining on ventrolateral view).
• 4.png (Figure 4 Bronchosyringeal cartilage).
• 5a.png (Figure 5A Syrinx muscle structures on dorsal view).
• 5b.png (Figure 5B Syrinx muscle structures on ventral view).
• 6a.png (Figure 6A Lovebird syrinx aged 2 months on lateral view).
• 6b.png (Figure 6B Lovebird syrinx aged 3 months on lateral view).
• 6c.png (Figure 6C Lovebird syrinx aged 4 months on lateral view).

Figshare: Gel electrophoresis result to determine Lovebird gender. https://doi.org/10.6084/m9.figshare.19248182.v1. ^49^

This project contains the following underlying data:

• Gel session 1.jpeg (Gender determination of sample number 1-12).
• Gel session 2.jpeg (Gender determination of sample number 13-24).
• Gel session 3.jpeg (Gender determination of sample number 25-33).

Figshare: Individually recorded Lovebird sounds in WAV format. https://doi.org/10.6084/m9.figshare.19327490.v1. ^50^

This project contains the following underlying data:

• ML1.wav (Sound recording for male lovebird sample No.1).
• ML2.wav (Sound recording for male lovebird sample No.2).
• ML3.wav (Sound recording for male lovebird sample No.3).
• ML4.wav (Sound recording for male lovebird sample No.4).
• FL1.wav (Sound recording for female lovebird sample No.1).
• FL2.wav (Sound recording for female lovebird sample No.2).
• FL3.wav (Sound recording for female lovebird sample No.3).
• FL4.wav (Sound recording for female lovebird sample No.4).

Figshare: Spectrogram analysis in respective bird sound. https://doi.org/10.6084/m9.figshare.19327856.v1. ^51^

This project contains the following underlying data:

• ML1.PNG (Spectrogram figure for male lovebird sample No.1).
• ML2.PNG (Spectrogram figure for male lovebird sample No.2).
• ML3.PNG (Spectrogram figure for male lovebird sample No.3).
• ML4.PNG (Spectrogram figure for male lovebird sample No.4).
• FL1.PNG (Spectrogram figure for female lovebird sample No.1).
• FL2.PNG (Spectrogram figure for female lovebird sample No.2).
• FL3.PNG (Spectrogram figure for female lovebird sample No.3).
• FL4.PNG (Spectrogram figure for female lovebird sample No.4).

### Reporting guidelines

Figshare: ARRIVE checklist for ‘An investigation of syrinx morphometry and sound frequency correlation during the chirping period in lovebirds ( *Agapornis fischeri*)’. https://doi.org/10.6084/m9.figshare.18394103.v2. ^52^

Data are available under the terms of the Creative Commons Attribution 4.0 International license (CC-BY 4.0).

## References

[1] Van der Zwan H Visser C Van der Sluis R : Plumage colour variations in the Agapornis genus: a review. *Ostrich.* 2019;90(1):1–10. 10.2989/00306525.2018.1540446

[2] Hanesia WI Hidayat B Sunarya U : Klasifikasi Suara Lovebird Dengan Metode Mel Frequency Cepstral Coefficient (mfcc) Dan Fuzzy Logic. *Proc. Eng.* 2015;2(2):2968.

[3] Suthers RA Zollinger SA : Producing song: the vocal apparatus. *Ann. N. Y. Acad. Sci.* 2004;1016(1):109–129. 10.1196/annals.1298.041 15313772

[4] Alonso RG Kopuchian C Amador A : Difference between the vocalizations of two sister species of pigeons explained in dynamical terms. *J. Comp. Physiol.* 2016;202(5):361–370. 10.1007/s00359-016-1082-3 27033354 PMC4979547

[5] Gündemir O Alpak H : Macroanatomic and histological examination of the trachea and syrinx in budgerigars and canaries. *Harran Üniv. Vet. Fak. Derg.* 2020;9(1):19–23. 10.31196/huvfd.652446

[6] Maksoud A Ibrahim A Hussein M : The Gross Anatomy of the Syrinx of Adult Male Domestic Fowl *Gallus gallus domesticus.* *J. Vet. Res. Med.* 2019;1(1):1–5.

[7] Riede T Goller F : Functional morphology of the sound-generating labia in the syrinx of two songbird species. *J. Anat.* 2010;216(1):23–36. 10.1111/j.1469-7580.2009.01161.x 19900184 PMC2807973

[8] Kriesell HJ Le Bohec C Cerwenka AF : Vocal tract anatomy of king penguins: morphological traits of two-voiced sound production. *Front. Zool.* 2020;17(1):1–11. 10.1186/s12983-020-0351-8 32021638 PMC6993382

[9] Soobramoney S Perrin M : A comparison of the alarm calls of five species of African lovebirds: genus Agapornis. *Trans. Royal Soc. South Africa.* 2014;69(1):9–18. 10.1080/0035919X.2014.890140

[10] Hongzhen Y Dongfeng L Jinchang J : The call development of *Psittacula agapornis.* *Shengwu Wuli Xuebao.* 2006;22(2):101–109.

[11] Casteleyn C Cornillie P Van Cruchten S : Anatomy of the upper respiratory tract in domestic birds, with emphasis on vocalization. *Anat. Histol. Embryol.* 2018;47(2):100–109. 10.1111/ahe.12336 29322535

[12] Mohamed R : Sexual dimorphism in the anatomical features of the Syrinx in the White Pekin ducks ( *Anas platyrhynchos*). *Int. J. Agric. Sci. Vet. Med.* 2017;5(5):78–85.

[13] Ladich F Winkler H : Acoustic communication in terrestrial and aquatic vertebrates. *J. Exp. Biol.* 2017;220(13):2306–2317. 10.1242/jeb.132944 28679789

[14] Federer WT : Randomization and sample size in experimentation. *Food and Drug Administration Statistics Seminar.* 1966; pp.2–3.

[15] Sigeman H Ponnikas S Hansson B : Whole-genome analysis across 10 songbird families within Sylvioidea reveals a novel autosome–sex chromosome fusion. *Biol. Lett.* 2020;16(4):20200082. 10.1098/rsbl.2020.0082 32315592 PMC7211462

[16] Gan HM Falk S Morales HE : Genomic evidence of neo-sex chromosomes in the eastern yellow robin. *GigaSci.* 2019;8(9):giz111. 10.1093/gigascience/giz111 31494668 PMC6736294

[17] Prapcoyo H Putra BPA Perwira RI : Implementation of Mel Frequency Cepstral Coefficient and Dynamic Time Warping For Bird Sound Classification. *In Conference SENATIK.* 2019;5(1):139–148. 10.28989/senatik.v5i0.326

[18] Abdel-Maksoud FM Hussein MM Hamdy A : Anatomical, Histological, and Electron Microscopic Structures of Syrinx in Male Budgerigars ( *Melopsittacus undulatus*). *Micros. Microanal.* 2020;26(6):1226–1235. 10.1017/S1431927620024460 33143802

[19] Alatrushi AN Naser AS : The Safety Profile of the Anesthetic Effect of Alfaxalone and its Interaction with Xylazine and Ketamine in Chick’s Model. *Maced. Vet. Rev.* 2021;44(2):203–209. 10.2478/macvetrev-2021-0025

[20] Riede T Olson CR : The vocal organ of hummingbirds shows convergence with songbirds. *Sci. Rep.* 2020;10(1):1–14. 32029812 10.1038/s41598-020-58843-5 PMC7005288

[21] Riede T Fisher JH Goller F : Sexual dimorphism of the zebra finch syrinx indicates adaptation for high fundamental frequencies in males. *PLoS One.* 2010;5(6):e11368. 10.1371/journal.pone.0011368 20614010 PMC2894075

[22] Kingsley EP Eliason CM Riede T : Identity and novelty in the avian syrinx. *Proc. Nat. Acad. Sci.* 2018;115(41):10209–10217. 10.1073/pnas.1804586115 30249637 PMC6187200

[23] Degrange FJ : A revision of skull morphology in Phorusrhacidae (Aves, Cariamiformes). *J. Vertebr. Paleontol.* 2020;40(6):e1848855. 10.1080/02724634.2020.1848855

[24] Gaban-Lima R Höfling E : Comparative anatomy of the syrinx in the tribe Arini (Aves: Psittacidae). *J. Morphol. Sci.* 2017;23(3):501–512.

[25] McInerney PL Lee MS Clement AM : The phylogenetic significance of the morphology of the syrinx, hyoid and larynx, of the southern cassowary, *Casuarius casuarius* (Aves, Palaeognathae). *BMC Evol. Biol.* 2019;19(1):1–18. 31881941 10.1186/s12862-019-1544-7 PMC6935130

[26] Abdel-Maksoud FM Hussein MM Hamdy A : Anatomical, Histological, and Electron Microscopic Structures of Syrinx in Male Budgerigars ( *Melopsittacus undulatus*). *Microsc. Microanal.* 2020;26(6):1226–1235. 10.1017/S1431927620024460 33143802

[27] Crosta L : Respiratory Diseases of Parrots: Anatomy, Physiology, Diagnosis and Treatment. *Vet. Clin. Exotic. Anim. Pract.* 2021;24(2):397–418. 10.1016/j.cvex.2021.01.005 33892893

[28] Monte A Cerwenka AF Ruthensteiner B : The hummingbird syrinx morphome: a detailed three-dimensional description of the black jacobin’s vocal organ. *BMC Zool.* 2020;5(1):1–15. 10.1186/s40850-020-00057-3

[29] Doğan GK Takaci I : Anatomy of respiratory system in poultry. *Vet. J. Mehmet. Akif. Ersoy. Univ.* 2018;3(2):141–147. 10.24880/maeuvfd.433946

[30] Kumar R Singh M : Bird fancier’s lung: clinical-radiological presentation in 15 cases. *Adv. Respir. Med.* 2015;83(1):39–44. 10.5603/PiAP.2015.0005 25577532

[31] Ibrahim IAA Hussein MM Hamdy A : Comparative morphological features of syrinx in male domestic fowl *Gallus gallus domesticus* and male domestic pigeon *Columba livia domestica*: A histochemical, ultrastructural, scanning electron microscopic and morphometrical study. *Micros. Microanal.* 2020;26(2):326–347. 10.1017/S1431927620000021 32000880

[32] Putranto HD Brata B Yumiati Y : Study on contour feathers growth of White-rumped Shama during fledgling phase. *In IOP Conf. Ser. Earth Environ. Sci.* 2021;788(1):012085. 10.1088/1755-1315/788/1/012085

[33] Al-badri AMS : Macroscopic study of syrinx in the common bulbul ( *Pycnontus barbatus*) and indigenous pigeon ( *Columba domestica*). *Al-Qadisiyah J. Vet. Med. Sci.* 2014;13(1):88–93. 10.29079/vol13iss1art284

[34] Zhang YS Takahashi DY Liao DA : Vocal state change through laryngeal development. *Nat. Commun.* 2019;10(1):1–12. 10.1038/s41467-019-12588-6 31597928 PMC6785551

[35] Walløe S Chakraborty M Balsby TJ : A Relationship between the Characteristics of the Oval Nucleus of the Mesopallium and Parrot Vocal Response to Playback. *Brain Behav. Evol.* 2021;96(1):37–48. 10.1159/000517489 34284396

[36] Ghosh R Mishra RC Choi B : Exposure to sound vibrations lead to transcriptomic, proteomic and hormonal changes in Arabidopsis. *Sci. Rep.* 2016;6(1):1–17. 10.1038/srep33370 27665921 PMC5036088

[37] Chiver I Schlinger BA : Sex-specific effects of testosterone on vocal output in a tropical suboscine bird. *Anim. Behav.* 2019;148(1):105–112. 10.1016/j.anbehav.2018.12.011

[38] Gahr M : How hormone-sensitive are bird songs and what are the underlying mechanisms?. *Acta Acust. United Acust.* 2014;100(4):705–718. 10.3813/AAA.918749

[39] Goller F : *Sound production and modification in birds–Mechanisms, methodology and open questions. Comparative Bioacoustics: An Overview.* Brown C Riede T , editors. Sharjah, United Arab Emirates: Bentham Science Publishers;2016;165–230.

[40] Kunduk M Vansant MB Ikuma T : The effects of the menstrual cycle on vibratory characteristics of the vocal folds investigated with high-speed digital imaging. *J. Voice.* 2017;31(2):182–187. 10.1016/j.jvoice.2016.08.001 27614383

[41] Tehrani MA Veney SL : Intracranial administration of the G-protein coupled estrogen receptor 1 antagonist, G-15, selectively affects dimorphic characteristics of the song system in zebra finches ( *Taeniopygia guttata*). *Develop Neurobiol.* 2018;78(8):775–784. 10.1002/dneu.22599 29675990

[42] Cornez G Shevchouk OT Ghorbanpoor S : Testosterone stimulates perineuronal nets development around parvalbumin cells in the adult canary brain in parallel with song crystallization. *Horm. Behav.* 2020;119(1):104643. 10.1016/j.yhbeh.2019.104643 31785283 PMC7065963

[43] Clark CJ Feo TJ : The Anna’s hummingbird chirps with its tail: a new mechanism of sonation in birds. *Proceed. Royal Soc. B Biol. Sci.* 2008;275(1637):955–962. 10.1098/rspb.2007.1619 PMC2599939 18230592

[44] Hayslette SE Mirarchi RE : Patterns of food preferences in mourning doves. *J. Wildlife Manag.* 2001;65(4):816–827. 10.2307/3803031

[45] Caras ML Remage-Healey L : Modulation of peripheral and central auditory processing by estrogens in birds. *Hear Horm.* 2016;57(4):77–99. 10.1007/978-3-319-26597-1_4

[46] Ko MC Van Meir V Vellema M : Characteristics of song, brain-anatomy and blood androgen levels in spontaneously singing female canaries. *Horm. Behav.* 2020;117(1):104614. 10.1016/j.yhbeh.2019.104614 31647922

[47] Purnama MTE Dewi CMS Dhamayanti Y : Syrinx morphometry and sound frequency evaluation. figshare. *Dataset.* 2022. 10.6084/m9.figshare.18382925.v4 PMC11109576 38779459

[48] Purnama MTE Dewi CMS Dhamayanti Y : Syrinx morphometry and topography. figshare. *Figure.* 2022. 10.6084/m9.figshare.18386744.v2

[49] Purnama MTE Dewi CMS Dhamayanti Y : Gel electrophoresis result to determine Lovebird gender. figshare. *Figure.* 2022. 10.6084/m9.figshare.19248182.v1

[50] Purnama MTE Dewi CMS Dhamayanti Y : Individually recorded Lovebird sounds in WAV format. figshare. *Media.* 2022. 10.6084/m9.figshare.19327490.v1

[51] Purnama MTE Dewi CMS Dhamayanti Y : Spectrogram analysis in respective bird sound. figshare. *Figure.* 2022. 10.6084/m9.figshare.19327856.v1

[52] Purnama MTE Dewi CMS Dhamayanti Y : ARRIVE report checklist: Syrinx morphometry and sound frequency evaluation during chirping period in Lovebirds (Agapornis fischeri). *figshare.* 2022. Online resource. 10.6084/m9.figshare.18394103.v2

