# Supplementary material for: An investigation of syrinx morphometry and sound frequency association in lovebirds ( Agapornis fischeri) chirps
Source: F1000Res. 2024 May 22;11:354. Originally published 2022 Mar 24. [Version 3] doi: 10.12688/f1000research.108884.3 (PMC11109576; doi:10.12688/f1000research.108884.3)
Supplement: Peer review history

# Reviewer report: Reviewer response for version 2

**DOI:** 10.5256/f1000research.143256.r266111

**Published:** 2024-05-10

**Authors:** Abdul Razak Alimon

**Affiliations:** 1 Department of Animal Science, Faculty of Agriculture, Putra Malaysia University, Serdang, Selangor, Malaysia

I have read the paper and in my opinion this paper is well written and has scientific merits.

 In Indonesia lovebirds are popular ornamental birds kept for their beautiful colours  and melodious chirps. In fact these birds are used in competition for their chirping ability. This study investigated the chirping frequency in relation to the morphology of the syrinx, which is where the voice originate. The description of  methodology was clear and  appropriate for this kind of study.  The results of this study were well presented and showed significant relationships between sound frequency and syrinx. 

 Overall, an interesting paper.

Is the work clearly and accurately presented and does it cite the current literature?

Yes

If applicable, is the statistical analysis and its interpretation appropriate?

Yes

Are all the source data underlying the results available to ensure full reproducibility?

Yes

Is the study design appropriate and is the work technically sound?

Yes

Are the conclusions drawn adequately supported by the results?

Yes

Are sufficient details of methods and analysis provided to allow replication by others?

Yes

Reviewer Expertise:

Animal nutrition and physiology.

I confirm that I have read this submission and believe that I have an appropriate level of expertise to confirm that it is of an acceptable scientific standard.

# Reviewer report: Reviewer response for version 2

**DOI:** 10.5256/f1000research.143256.r161020

**Published:** 2023-01-23

**Authors:** Abdulmojeed Yakubu

**Affiliations:** 1 Department of Animal Science/Centre for Sustainable Agriculture and Rural Development, Faculty of Agriculture, Nasarawa State University, Lafia, Nigeria

I have read the revised version of the manuscript entitled 'An investigation of syrinx morphometry and sound frequency association during the chirping period in lovebirds ( *Agapornis fischeri*)'. It has been substantially improved by the authors. Therefore, the manuscript can be accepted for indexing in its current form.

Is the work clearly and accurately presented and does it cite the current literature?

Yes

If applicable, is the statistical analysis and its interpretation appropriate?

Partly

Are all the source data underlying the results available to ensure full reproducibility?

Yes

Is the study design appropriate and is the work technically sound?

Yes

Are the conclusions drawn adequately supported by the results?

Partly

Are sufficient details of methods and analysis provided to allow replication by others?

Yes

Reviewer Expertise:

Animal Breeding and Genetics

I confirm that I have read this submission and believe that I have an appropriate level of expertise to confirm that it is of an acceptable scientific standard.

# Reviewer report: Reviewer response for version 1

**DOI:** 10.5256/f1000research.120324.r144637

**Published:** 2022-08-02

**Authors:** Abdulmojeed Yakubu

**Affiliations:** 1 Department of Animal Science/Centre for Sustainable Agriculture and Rural Development, Faculty of Agriculture, Nasarawa State University, Lafia, Nigeria

**Abstract:**

 Background:

- I thought the location of study will be a thing of interest.

Methods:

- A brief description of the statistical analysis is required under Methods.
- This sentence appears incomplete: ‘On the other hand, the tympaniform membranes lateral dexter (p=0,02) and sinister (p=0,05) in females showed wider compared to male’
- You gave regression equations without the corresponding correlation values, except you want to replace ‘correlation’ with ‘association’.

Conclusions:

- Please try and conclude in the context of the biological implication of your findings.

**Introduction:**

- Please expatiate on ‘Lovebirds contest’.

**Methods:**

 Experimental design:

- Try and separate experimental design from PCR part of the study.
- Provide a reference for the Federer’s formula: t(n-1) ≥ 15, and why is it adopted in this study considering the sample size of only 24 lovebirds?
- What gel and dye were used for the PCR?
- ‘Of the 33 samples’.  There is a need to elaborate on this.
- Briefly describe the term ‘blinding’.

Methylene blue staining:

- How soon does lethal dose (LD50) have effect on the bird?

Statistical analysis:

- Did you test for the homogeneity of variance and normality of your data?
- You are mixing up Correlation and Regression (Association). Although the two has to do with ‘Relationship’, they provide different values.

**Results:**

 Sound frequency evaluation:

- You should have tested sound frequency for other ages as it would have provided additional information for comparison.

**Discussion:**

- Briefly discuss syrinx morphometrics in the context of adaptation before relating with sound frequency.

**Conclusions:**

- Try and beef up this section especially as it relates to biological implication and future study.

**References**

- Please revisit for accuracy and correct formatting.

NB: There is a need for language editing of the entire text.

Is the work clearly and accurately presented and does it cite the current literature?

Yes

If applicable, is the statistical analysis and its interpretation appropriate?

Partly

Are all the source data underlying the results available to ensure full reproducibility?

Yes

Is the study design appropriate and is the work technically sound?

Yes

Are the conclusions drawn adequately supported by the results?

Partly

Are sufficient details of methods and analysis provided to allow replication by others?

Yes

Reviewer Expertise:

Animal Breeding and Genetics

I confirm that I have read this submission and believe that I have an appropriate level of expertise to confirm that it is of an acceptable scientific standard, however I have significant reservations, as outlined above.

# Response

**Published:** 2023-01-15

**Authors:** Muhammad Thohawi Elziyad Purnama

**Affiliations:** Airlangga University, Indonesia

Dear Reviewer,

 Thank you for your valuable comments and suggestions on the manuscript entitled:

**"An investigation of syrinx morphometry and sound frequency association during the chirping period in lovebirds (Agapornis fischeri)"**

 We welcome feedback. We have made modifications to the study on the following points:

**Background:**

 I thought the location of study will be a thing of interest.

**Response:** We have added the location and study period to the background abstract and methods section.

**Methods:**

 A brief description of the statistical analysis is required under Methods.

**Response:** We have added a brief description of the statistical method to the abstract.

 This sentence appears incomplete: ‘On the other hand, the tympaniform membranes lateral dexter (p=0,02) and sinister (p=0,05) in females showed wider compared to male’

**Response:** We have modified the interpretation of these results.

 You gave regression equations without the corresponding correlation values, except you want to replace ‘correlation’ with ‘association’.

**Response:** We have revised that the association test was used in the present study.

**Conclusions:**

 Please try and conclude in the context of the biological implication of your findings.

**Response:** We have modified the conclusions according to crucial information on syrinx morphometry and biologic implications.

**Introduction:**

 Please expatiate on ‘Lovebirds contest’.

**Response:** We have added crucial information about lovebird contests and information gaps from this study.

**Methods:**

 Experimental design:

 Try and separate experimental design from PCR part of the study.

**Response:** We have separated the experimental design section from the sex determination method.

 Provide a reference for the Federer’s formula: t(n-1) ≥ 15, and why is it adopted in this study considering the sample size of only 24 lovebirds?

**Response:** We have added references and reasons why this formula was implemented in determining the sample for this study.

 What gel and dye were used for the PCR?

**Response:** We have added manufacturing information for gel products and dyes used.

 ‘Of the 33 samples’.  There is a need to elaborate on this.

**Response:** We have added a description of why the number of birds was collected, then determined the sex as a qualification for the sample studied.

 Briefly describe the term ‘blinding’.

**Response:** We have described the randomization and blinding stages of the sample.

 Methylene blue staining:

 How soon does lethal dose (LD50) have effect on the bird?

**Response:** We have added information on the onset of action LD50 at the euthanasia stage.

 Statistical analysis:

 Did you test for the homogeneity of variance and normality of your data?

**Response:** We added a brief description of the Shapiro-Wilk test for homogeneity analysis in the present study.

 You are mixing up Correlation and Regression (Association). Although the two has to do with ‘Relationship’, they provide different values.

**Response:** We have revised that the association test was used in the present study.

**Results:**

 Sound frequency evaluation:

 You should have tested sound frequency for other ages as it would have provided additional information for comparison.

**Response:** A limitation of our study was not evaluating the frequency of birdsong at all ages due to the full rearing period up to 4 months of age prior to euthanasia. However, we will use this design for further studies so that we can provide more comprehensive information and compare it to other ages.

**Discussion:**

 Briefly discuss syrinx morphometrics in the context of adaptation before relating with sound frequency.

**Response:** We have added a brief description of the adaptive context of the syrinx morphometrics prior to its physiological function in producing sound vibrations.

**Conclusions:**

 Try and beef up this section especially as it relates to biological implication and future study.

**Response:** We have modified the conclusions according to crucial information on syrinx morphometry and biological implications.

**References**

 Please revisit for accuracy and correct formatting.

**Response:** We have revised and reformatted the references according to the guidelines.
